# The Immune Landscape and Prognostic Immune Key Genes Potentially Involved in Modulating Synaptic Functions in Prostate Cancer

**DOI:** 10.3389/fonc.2020.01330

**Published:** 2020-08-14

**Authors:** Sha Zhu, Xu Han, Xianli Qiao, Shengxian Chen

**Affiliations:** Key Laboratory of Tumor Immunity, Center of Infection and Immunization, Department of Immunology, School of Basic Medical Sciences, Zhengzhou University, Zhengzhou, China

**Keywords:** tumor-infiltrating immune cells, immune landscape, prostate cancer, immunological synapse, prognostic

## Abstract

**Background:** Increasing evidence has indicated an association between differentially expressed genes (DEGs) in tumor-infiltrating immune cells (TIICs) and clinical outcome. The aim of this research is to investigate the influence of tumor microenvironment on the gene expression profile of TIICs and to identify their potential markers for modulating immune cell function in prostate cancer.

**Methods:** In our research, CIBERSORT algorithm was utilized to calculate the proportion of the TIICs in 164 tumor and 18 control samples from The Cancer Genome Atlas cohort. The differential expression analysis was conducted using R, and then the functional and the pathway enrichments of the DEGs were analyzed using Database for Annotation, Visualization, and Integrated Discovery, followed by integrated regulatory network analysis.

**Results:** As a result, nTreg, B cells, Th1, and DC cells were significantly increased, accompanied by largely decreased NK and NKT cells. The expressed immune-related gene correlation analysis showed that the signature gene expression extent of CD8 T cells was positively associated with CD4 memory activated T cells but negatively correlated with that of CD4 memory resting T cells. In addition, a total of 128 differentially expressed genes were identified. CytoHubba analysis obtained six hub genes, of which three prognostic-associated potential key molecules including CAV1, FLNA, and VCL were mainly involved in biological processes associated with the regulation of organic substance and synaptic connections.

**Conclusions:** This study provides a comprehensive understanding of the landscape of TIICs and the roles of the hub genes which may be valuable markers in prostate cancer diagnosis and immunotherapy.

## Introduction

Prostate cancer (PCa) is one of the most frequently diagnosed neoplasm in many parts of the world, with an estimated 174,650 new cases and 31,620 deaths occurring in 2019 in the United States (1). Radical prostatectomy represents the standard treatment for localized PCa; disease recurrence after surgery remains a major clinical challenge. Previous studies have shown that adjuvant chemotherapy could improve the survival rate in these patients. Especially for advanced PCa, hormonal deprivation therapy can temporarily inhibit tumor progression through the androgen receptor signaling pathway, but this function is only effective for ~2 years. Therefore, it is clear that new immunotherapeutic trials and prognostic markers of tumor-infiltrating immune cells (TIICs) are needed for better clinical management.

It is well-known that the immune system has a vital role in controlling tumor progression. TIICs are essential components of the tumor microenvironment (TME) and most likely to be used as drug targets to improve the survival rate of patients. Intratumoral immune cells are increasingly recognized as responsible for antitumor immune responses inducing tumor growth, invasion, angiogenesis, and metastasis, which are associated with clinical outcomes (2). In the TME, pre-existing lymphocytes can recognize abnormally expressed neoantigens and play an important role in tumor regression (3). CD8 T cells in malignant tumors have been associated with better survival in breast cancer (BC), small cell lung carcinoma (SCLC), carcinomas of colon, bile duct, urothelium, and esophagus, as well as follicular lymphoma and uveal melanoma (4–11). Studies indicated that regulatory T (Treg) cells correlated with suppressing a great number of immune cells, including T cells (both CD8 and CD4 T cells), natural killer T (NKT) cells, natural killer (NK) cells, dendritic cells (DCs), Th17, B cells, and monocytes (12–18). Accumulating evidence confirmed the prognostic value of TIICs for many tumors, and it has been documented that high intratumoral immune cell intensity is associated with a better outcome in SCLC and BC (19, 20).

Previous studies have primarily used flow cytometry or immunohistochemistry (IHC) to assess the composition of infiltrating immune cells in tumors. Flow cytometry is usually based on several marker proteins, which is limited by the number of fluorescence channels, and only a few immune cell types can be evaluated at once by IHC. A systems biology tool, CIBERSORT, has been developed, which employs deconvolution of bulk gene expression data and a sophisticated algorithm for the quantification of many immune cell types in heterogeneous tumor samples (21). The type and the quantity of primordial cells in the cell mixtures of the tissue can be reconstructed and have been successfully verified by FACS. Using characteristic marker genes, CIBERSORT can quantify the relative scores of each immune cell type, which has been used in various malignant tumors (e.g., breast cancer and colon cancer) (22, 23). Importantly, the significance of the expression of immunoregulatory proteins such as PD-1/PD-L1 and CTLA4 has gained increasing attention in the scientific community in recent years. However, only few other immune cell-related molecular markers are known to be used for survival prediction or cancer treatment (24). Therefore, identification of key molecules involving PCa outgrowth is urgently required, which may successively provide novel prognostic markers and/or novel targeted therapies in patients with PCa.

In this work, the RNA-seq data of 182 samples, including 164 prostate tumors and 18 normal tissues based on The Cancer Genome Atlas (TCGA) database, were analyzed. The results might provide clues for a better understanding of the gene expression profiles involved in modulation of the phenotype of TIICs and to identify prognostic and predictive immune cell-related differentially expressed genes (DEGs) in PCa, which may offer clinical benefit to control and suppress tumor progression.

## Subjects and Methods

### Data Acquisition and TIIC Evaluation

The gene-level RNA-Seq data of 164 primary prostate adenocarcinoma and 18 relevant normal samples were retrieved from the TCGA data portal (https://tcga-data.nci.nih.gov/tcga/). The standard gene expression data were included to evaluate the relative proportions of 22 TIIC subpopulations by utilizing the CIBERSORT algorithm. CIBERSORT is a deconvolution algorithm based on the gene expression data to characterize immune cell composition using Monte Carlo sampling, which provided a measure of confidence in the result (21). These immune cell components included naïve B cells, plasma cells, memory B cells, CD8 T cells, naïve CD4 T cells, resting CD4 memory T cells, activated CD4 memory T cells, Tregs, follicular helper T cells (Tfh), γδ T cells, resting NK cells, activated NK cells, activated mast cells, resting mast cells, M0 macrophages, M1 macrophages, M2 macrophages, resting dendritic cells, activated dendritic cells, monocytes, neutrophils, and eosinophils. The samples with *P* < 0.05 were included.

### Differential Expression Analysis

The gene expression data were normalized by the R package “limma” based on the Benjamini and Hochberg procedure (25) to screen DEGs by performing background correction, data normalization, conversion of original data, and expression calculation. Genes differentially expressed between tumor and relevant controls were defined using fold change >2 and *P* < 0.05 as cutoff criteria. Subsequently, the probes were annotated based on platform annotation files, and the probes without matching gene symbols were removed. For the different probes mapped to one and the same gene symbol, the average value of the probes was obtained as the gene expression value.

### Functional and Pathway Enrichment Analysis

Database for Annotation, Visualization and Integrated Discovery (26) was used for Gene Ontology (GO) enrichment analysis. The DEGs of TIICs of prostate cancer patients were screened for functional enrichment. GO analysis was used to predict the degree of enrichment and the potential functions of the DEGs in biological processes, cellular components, and molecular functions. In addition, the Kyoto Encyclopedia of Genes and Genomes (KEGG) database was used for a systematic analysis of differences in gene functions. Using the database for annotation, visualization, and integrated discovery tool (version 6.7, https://david-d.ncifcrf.gov/) (27), the up-regulated and the down-regulated genes were conducted with GO functional and KEGG pathway enrichment analyses, respectively. The terms with *P*-value < 0.05 and count (the number of enriched genes) ≥2 were considered as the cutoff criterion.

### PPI Network and Module Analyses

The Search Tool for the Retrieval of Interacting Genes (STRING, version 10.0, http://www.string-db.org/) (28) is an online database providing experimental and predicted protein–protein interaction (PPI) information. The PPI pairs among the proteins encoded by the DEGs were predicted and analyzed by the STRING database. The parameter was set with a medium confidence score >0.4. Then, PPI network was constructed using Cytoscape software (version 3.2.0, http://www.cytoscape.org) (29) to visualize the interaction of the up-regulated and the down-regulated genes (30). Subsequently, the degree centrality of the nodes in the PPI network was calculated by the CytoHubba plug-in (31) in Cytoscape software, the parameter of which was set as without an expanded subnetwork. The nodes with higher degrees were identified as the hub proteins (32). Additionally, the KEGG pathway enrichment analysis for nodes in the significant modules was performed using MATHT tool.

### Immunofluorescence Evaluation

Immunofluorescence staining was carried out with 4-μm-thick sections of formalin-fixed, paraffin-embedded tumor tissues from clinical patients with prostate cancer. Following deparaffinization and rehydration of the tissue sections, antigen retrieval was performed by microwaving in 10 mM citrate buffer (pH 6.0). Primary anti-CD4 antibody (Agilent Technologies) was applied at 1:50 dilution. Alexa Fluor 594 or FITC (BioLegend, San Diego, CA, USA) was used, at a concentration of 1.5 μg/ml, as a secondary antibody for 1 h, and the nuclei were counterstained using 4′-6-diamidino-2-phenylindole (Sigma, Portland, OR, USA) for 5 min. After mounting, the sections were observed under an Olympus BX51 microscope at a magnification of ×200. At least four sections of tumor tissue were used for quantitative evaluation.

### Statistics

The *P*-values for the experimental data were generated using a two-tailed Student's *t*-test with unequal variance (or a χ^2^ test where indicated). A *P-*value of less than 0.05 was considered as significant. Data are shown as mean ± SD.

## Results

### Distribution and Correlation of TIICs in Prostate Cancer

**Figure 1:**
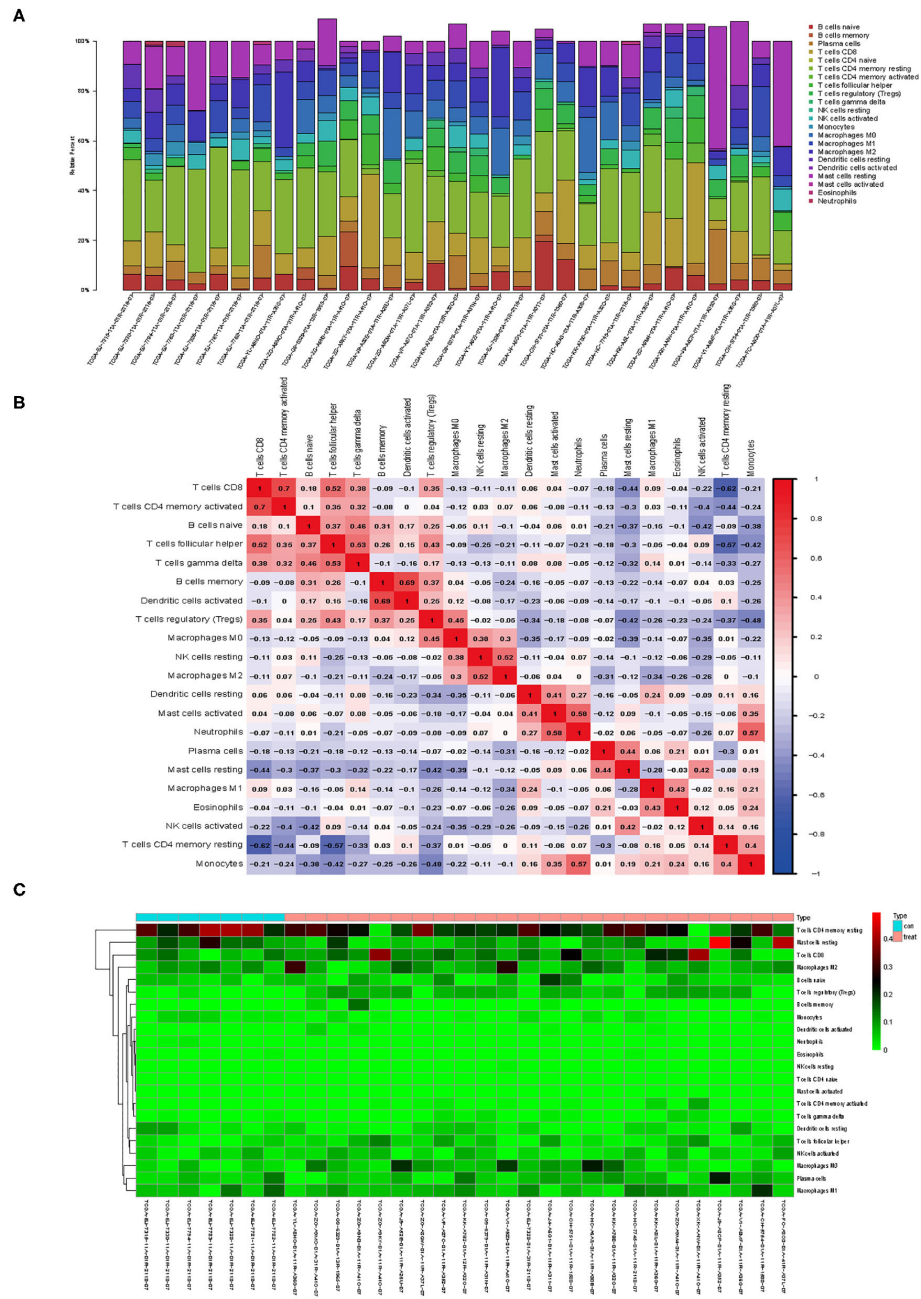
Landscape of immune cell infiltration in prostate cancer (PCa) tissues. **(A)** Bar charts of 22 immune cell proportions in PCa and normal tissues. **(B)** Correlation matrix of all immune cell proportions. **(C)** Heat map of the immune cell proportions between PCa and normal tissues. The standardized immune cell infiltration levels are depicted by the color gradient: green, low infiltration ratio; red, high infiltration ratio. Values in black represent a ratio equal to 0. Result visualization was performed by using R package ggplot2 (version 3.1.0). Correlation analysis and visualization were performed by using R package corrplot (version 0.84).

CIBERSORT, a deconvolution algorithm method based on gene expression, was used to predict the fractions of multiple immune cell types in the gene expression profiles of admixtures. The cellular composition can be estimated based on standardized gene expression data, which indicate the abundances of specific cell types. In this study, we explored TIICs in PCa tissue by the CIBERSORT algorithm. Based on the criteria of *P* < 0.05, 31 samples were included. Figure 1A shows the proportions of immune cells in each PCa sample in different colors, and the lengths of the bars in the bar chart indicate the levels of the immune cell populations. CD4 memory resting T cells had a significantly low percentage in TIICs (*P* < 0.001).

Figure 1B indicates the correlation between these differentially expressed types of immune cells. The result revealed that CD8 T cells were positively correlated with CD4 memory activated T cells but negatively correlated with CD4 memory resting T cells in PCa. As shown in the chart in Figure 1C, we identified that regulatory T cells had a relatively high percentage (*P* < 0.001), but CD4 memory resting T cells had a significantly low percentage in TIICs (*P* < 0.001).

### DEGs and Significance of Extensive Infiltration of TIICs

**Figure 2:**
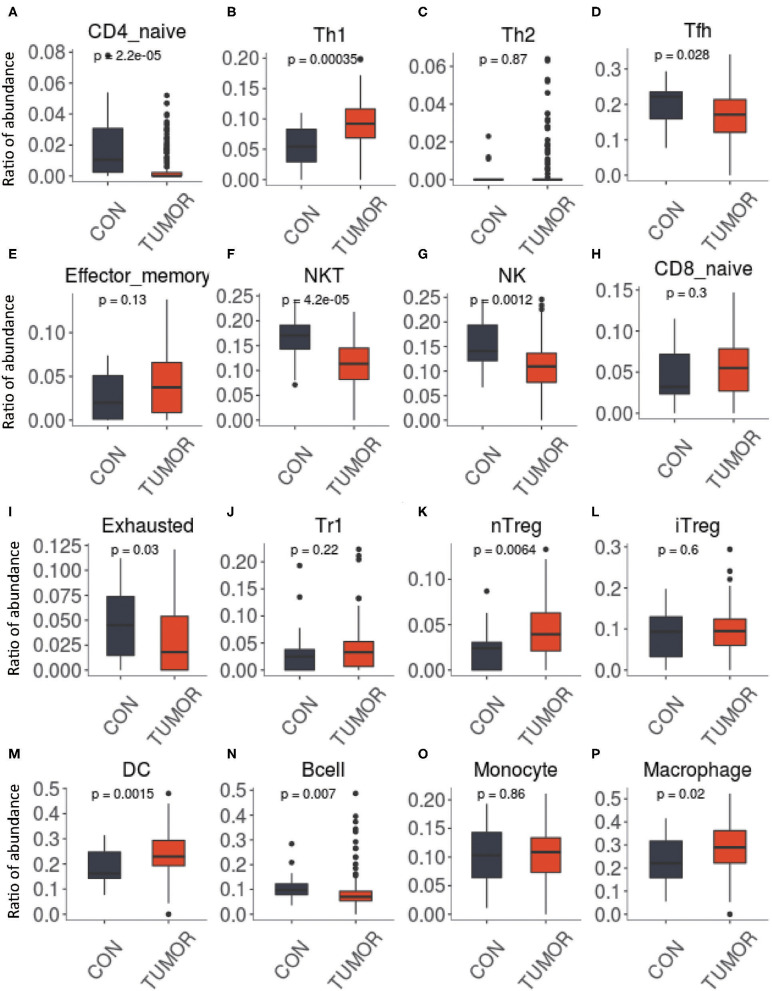
Immune cell abundance was analyzed and checked by immune cell abundance identifier (ImmuCellAI) in groups between prostate cancer (PCa) and normal tissues. **(A)** CD4 naive, **(B)** CD8 naive, **(C)** Th1, **(D)** Th2, **(E)** effector memory, **(F)** NKT, **(G)** NK, **(H)** Tfh, **(I)** exhausted, **(J)** Tr1, **(K)** nTreg, **(L)** iTreg, **(M)** DC, **(N)** B cell, **(O)** monocyte, and **(P)** macrophage. Expression data used in ImmuCellAI were collated by using R package tidyverse (version 1.3.0). R package ggpubr (version 0.2.4) was used for *t*-test.

The DEGs were identified by the Robust MultiArray Averaging method in R package. Threshold |logFC| >1 and *P* < 0.05 were used as criteria for comparison (33). Genes differentially expressed of TIICs screened based on the database CellMarker (http://biocc.hrbmu.edu.cn/CellMarker/) between PCa and the controls were defined for further study. The presence of TIIC profiles was reanalyzed by applying IMMUCELL AI criteria. The results showed that the intratumoral TIIC profiles were significantly enriched in nTreg (*P* = 0.0064), Th1 (*P* < 0.001), and DC (*P* = 0.0015), but the CD4 naive (*P* < 0.001), NK (*P* = 0.0012), and NKT cells (*P* < 0.001) were significantly decreased in the PCa tissues (Figures 2A–P).

### Functional Annotation and Pathway Enrichment

The enriched GO terms and KEGG pathways for the TIIC-related DEGs were identified (26). As shown in Table 1, the top three biological process terms associated with the up-regulated and down-regulated DEGs were involved in the response to organic substance, oxidative stress, and hormone stimulus. In terms of cellular components, the DEGs were mostly associated with extracellular matrix and basolateral plasma membrane. Molecular function analysis indicated that the DEGs were mainly enriched in mitogen-activated protein kinase phosphatase activity and cytoskeletal protein binding. A subsequent KEGG pathway enrichment analysis revealed that the functions of the DEGs were primarily enriched in focal adhesion, nicotinate and nicotinamide metabolism, and phosphatidylinositol signaling system. The terms with *P*-value < 0.05 and count (the number of enriched genes) ≥2 were considered as the cutoff criterion.

**Table 1:** GO functional and KEGG pathway enrichment analysis of DEGs.

**ID**	**Terms**	**Count**	***P*-value**	**Genes**
**Biological process**				
GO:0010033	Response to organic substance	15	1.68E-04	CAV1, NR3C1, FOS, DUSP4, CD38, SDC1, ID2, BTG2, DUSP1, CCND2…
GO:0006979	Response to oxidative stress	8	1.86E-04	PYCR1, FOS, CD38, SDC1, DUSP1, BCL2, PDLIM1, PRNP
GO:0009725	Response to hormone stimulus	10	1.36E-03	FOS, CD38, CAV1, SDC1, DUSP1, BTG2, CCND2, GATA3, BCL2, TGFBR3
GO:0001666	Response to hypoxia	6	2.88E-03	CD38, CAV1, ATP1B1, BCL2, PDLIM1, DDIT4
GO:0051591	Response to cAMP	4	3.45E-03	FOS, SDC1, DUSP1, CCND2
**Cellular components**				
GO:0005578	Proteinaceous extracellular matrix	10	3.47E-04	COCH, CTHRC1, COL9A2, LGALS3BP, FBLN1, CD248, LGALS1, DCN, SPON2, ANXA2
GO:0016323	Basolateral plasma membrane	8	4.92E-04	CAV1, ATP1B1, SDC1, RHCG, ANK2, LAYN, ACTN1, VCL
GO:0031012	Extracellular matrix	10	6.00E-04	COCH, CTHRC1, COL9A2, LGALS3BP, FBLN1, CD248, LGALS1, DCN, SPON2, ANXA2
GO:0044421	Extracellular region part	16	2.17E-03	COCH, LGALS1, CD248, DCN, ANXA2, DKK3, COL9A2, FBLN1, CXCL13, CFH…
GO:0016324	Apical plasma membrane	6	2.22E-03	ANXA6, CAV1, ATP1B1, RHCG, ANK2, MAL
**Molecular functions**				
GO:0033549	MAP kinase phosphatase activity	4	9.97E-05	DUSP5, DUSP4, DUSP2, DUSP1
GO:0008092	Cytoskeletal protein binding	13	2.71E-04	SDC1, KLHL5, RHCG, SVIL, CAPG, ACTN1, CNN2, PRNP, FLNA, VCL…
GO:0008138	Protein phosphatase activity	4	3.93E-03	DUSP5, DUSP4, DUSP2, DUSP1
GO:0003779	Actin binding	8	9.37E-03	KLHL5, SVIL, CAPG, ACTN1, CNN2, FLNA, VCL, SYNPO
GO:0016791	Phosphatase activity	7	9.37E-03	DUSP5, INPP1, DUSP4, DUSP2, DUSP1, INPP5D, NT5E
**KEGG pathways**				
hsa04510	Focal adhesion	7	1.32E-02	CAV1, CCND2, BCL2, ACTN1, FLNA, PRKCB, VCL
hsa00760	Nicotinate and nicotinamide metabolism	3	2.28E-02	CD38, QPRT, NT5E
hsa04070	Phosphatidylinositol signaling system	4	3.56E-02	INPP1, PIP5K1B, INPP5D, PRKCB
hsa04010	MAPK signaling pathway	7	4.53E-02	DUSP5, FOS, DUSP4, DUSP2, DUSP1, FLNA, PRKCB
hsa04666	Fc gamma R-mediated phagocytosis	4	6.59E-02	MARCKSL1, PIP5K1B, INPP5D, PRKCB

### Analyzing the Modules of the Networks

**Figure 3:**
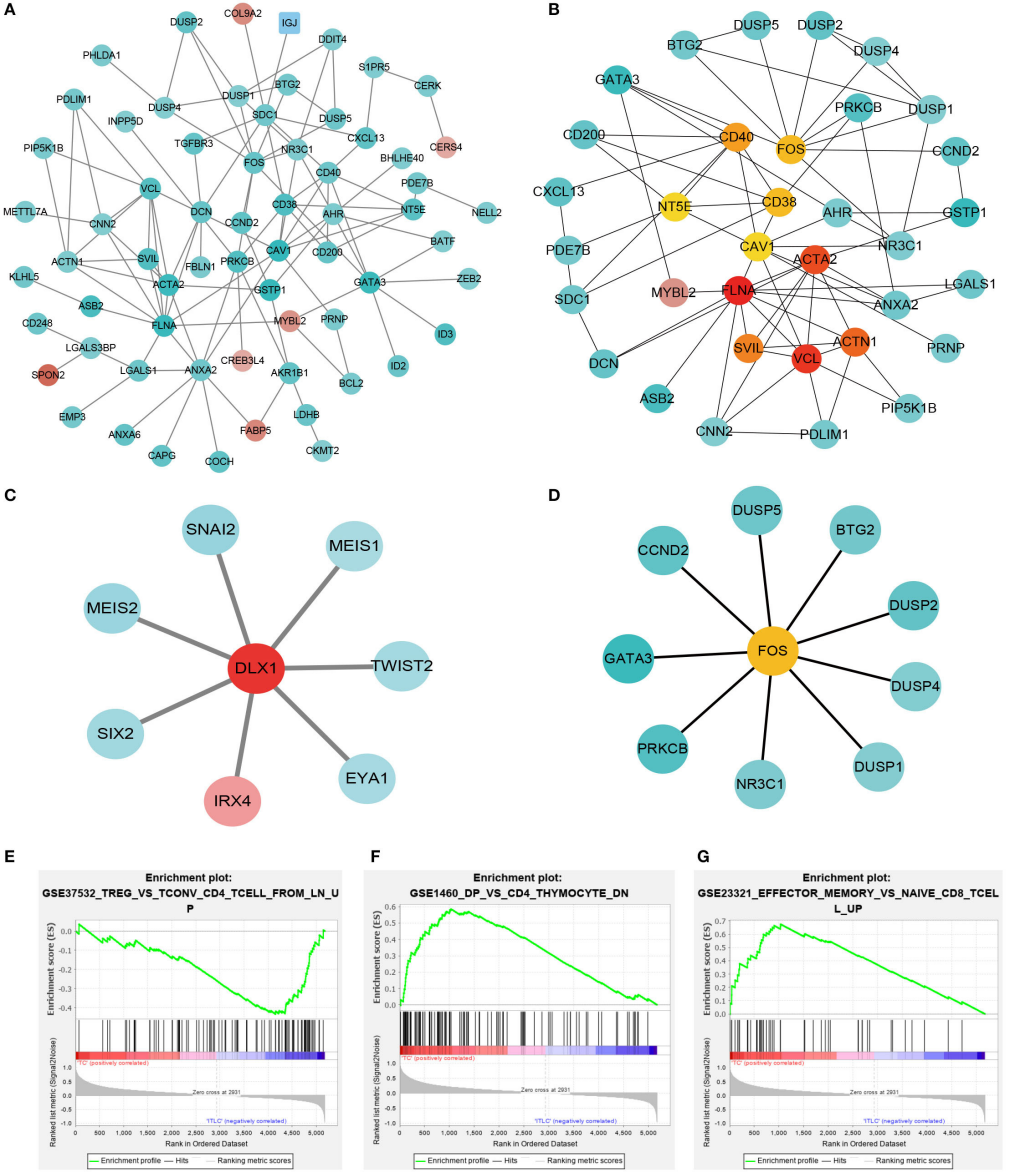
Protein–protein interaction networks and Gene Set Enrichment Analysis. **(A)** Network of significant proteins from the tumor-infiltrating immune cell (TIIC)-associated differentially expressed genes. Red and green intensities indicate the degree of up-regulation and down-regulation, respectively. **(B)** Significant hub proteins extracted by Edge Percolated Component algorithm from network A. **(C)** TIIC-associated transcription factor DLX1-centered regulatory network. **(D)** TIIC-associated transcription factor FOS-centered regulatory network. **(E)** Enrichment of genes in TREG_VS_TCONV_CD4_TCELL_FROM_LN_UP. **(F)** Enrichment of genes in DP_VS_CD4_THYMOCYTE_DN. **(G)** Enrichment of genes in EFFECTOR_MEMORY_VS_NAIVE_CD8_TCELL_UP.

To investigate the gene expression module of TIIC results in the influence of tumor microenvironment, PPI network was generated using the significantly expressed immune cell-related DEGs (Figure 3A). To further identify the hub gene and the key pathways, edge percolated component and shortest path analyses were conducted by CytoHubba plug-in in Cytoscape. The most significant modules composed of 10 nodes were screened out from the PPI networks (Figure 3B). In addition, to characterize the properties of the key nodes based on the analysis of the PPI network, we initially selected first-stage nodes associated with the transcription factor DLX1 and FOS to identify the potential candidate markers which may exert a significant influence on the biological function of the TIICs (Figures 3C,D). Moreover, Gene Set Enrichment Analysis (GSEA) was implemented between control and PCa groups. The results revealed that the gene sets were mainly enriched in regulatory T cells (Treg) (Figure 3E); however, few gene sets were distributed in double-positive (DP) and effector memory T cells of the PCa tissues (Figures 3F,G). These analyses reflect a negative regulation and a cold tumor microenvironment of PCa.

### The Expression Profile of Immune DEGs

**Figure 4:**
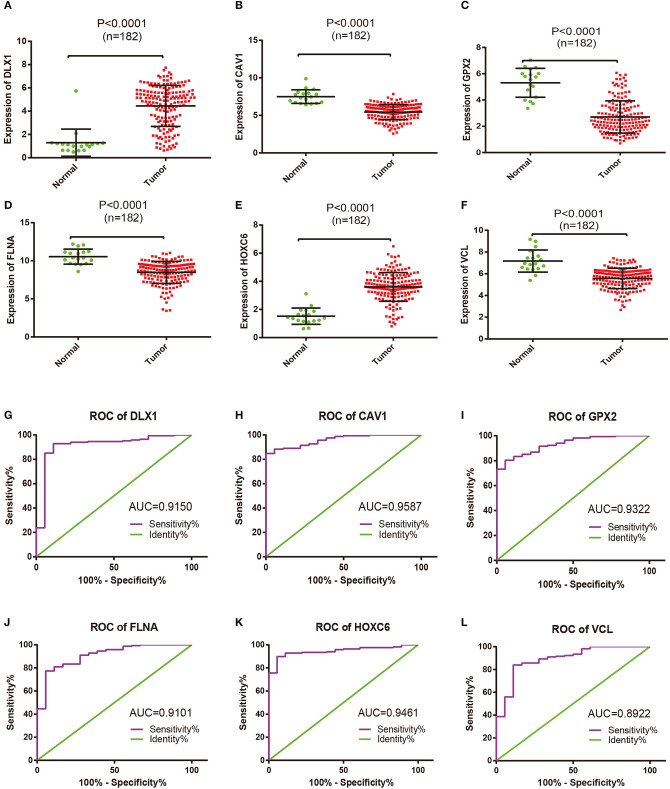
Comparable evaluation of the expression patterns and receiver operating characteristic (ROC) curves of six differentially expressed hub genes between PCa and control samples. Expression levels of **(A)** DLX1, **(B)** CAV1, **(C)** GPX2, **(D)** FLNA, **(E)** HOXC6, and **(F)** VCL; each dot represents one patient. Mean values and standard deviations were calculated for each patient group and compared using one-way ANOVA. ROC curves of **(G)** DLX1, **(H)** CAV1, **(I)** GPX2, **(J)** FLNA, **(K)** HOXC6, and **(L)** VCL.

The expression pattern of six differentially expressed hub genes including DLX1, CAV1, GPX2, FLNA, HOXC6, and VCL in all samples between two groups was analyzed (shown in Figures 4A–F). The results demonstrated that the mRNA expression levels of DLX1 and HOXC6 were significantly increased, whereas the expression levels of the other genes were remarkably decreased in TIICs compared with those in the controls. Receiver operating characteristic analysis was then performed for all of the hub genes (Figures 4G–L), and area under the curve represented the set of all possible statistical tests of the microarray data with equal probability for a true positive and a false positive result based on each decision threshold value. Of these hub genes, the expression levels of HOXC6 and CAV1 in PCa and controls were shown to be in good consistency.

### Identification and Evaluation of Prognosis-Associated Key Molecules

**Figure 5:**
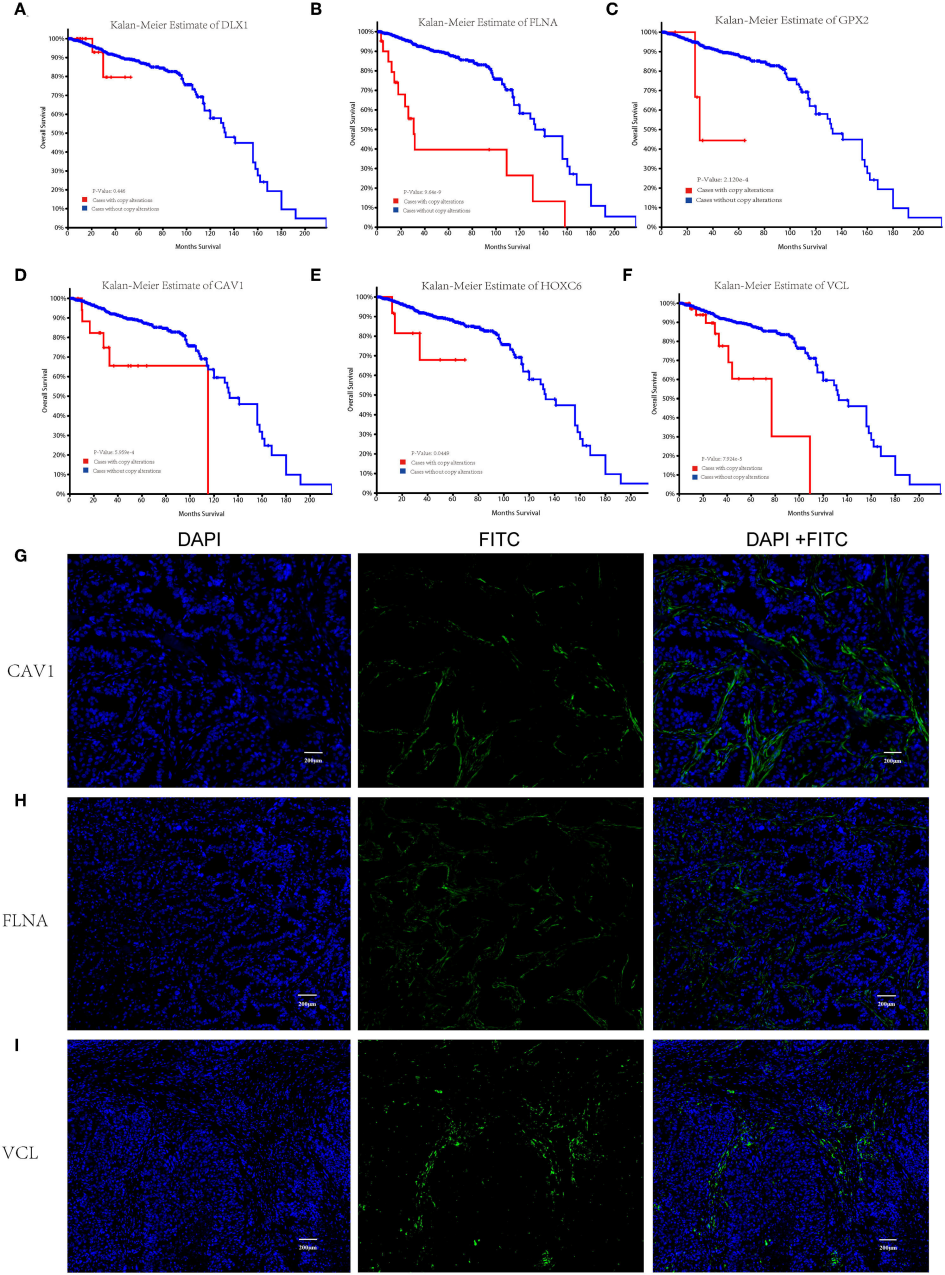
Kaplan–Meier overall survival analysis of the hub genes in prostate cancer (PCa) patients and immunofluorescence image of the key molecules CAV1, FLNA, and VCL. Overall survival curves for **(D)** DLX1, **(E)** FLNA, **(F)** GPX2, **(G)** CAV1, **(H)** HOXC6, and **(I)** VCL in PCa patients. Red lines: cases with copy alteration; blue lines: cases without copy alteration. *p* < 0.05 is considered as significant. Immunofluorescence labeling for **(A)** DLX1, **(B)** FLNA, and **(C)** CAV1 in clinical representative samples of PCa patients.

Kaplan–Meier survival analysis was conducted to identify the prognostic values of the hub genes with regard to the overall survival of PCa patients from the TCGA cohort. The results demonstrated that four hub molecules including FLNA, GPX2, CAV1, and VCL were significantly associated with overall survival in PCa patients (Figures 5A–F). The expression characteristics of the synaptic function-associated potential key molecules VCL, FLNA, and CAV1 were further identified by immunofluorescence staining in clinical samples of PCa patients, and it was observed that these proteins were particularly expressed in TIICs of the TME. However, they are scarcely found in tumor cells (Figures 5G–I). These key molecules differentially expressed in the TIICs may have an important clinical value in PCa diagnosis and immunotherapy.

## Discussion

Immune cells are the fundamental ingredients of the tumor microenvironment. Except for malignant neoplastic cells, TIICs including CD4 T cells, CD8 T cells, Treg, macrophage, DC, and NK cells also play important roles in shaping the tumor microenvironment. Those TIIC or bioactive molecule-mediated interactions can exert inhibitory effects on the malignant cells, whereas during tumor progression the tumor cells may exploit immune cells or relative molecules to circumvent these inhibitory signals (34). A growing number of studies have shown that the TIICs can be used to predict the clinical outcome and the treatment response of various tumors (35, 36). Actually, increasing evidence indicated that increased active T cells and memory T-cell infiltration are closely related to a favorable prognosis (37), but immune-suppressive regulatory T cells are the opposite (38).

To determine the prognostic value of TIICs, we used CIBERSORT for deconvolution of global gene expression data to define the immune cell landscape in PCa. The results showed that most of the enriched infiltrates were nTreg, Th1, DC, and macrophages. The nTreg cells cover subsets of iTreg cells, inducible co-stimulator (ICOS) Treg cells, Tr1 cells, and IL-17-producing Treg cells. These Treg cells share some common features, such as Foxp3 expression (Tr1 cells are the exception), and release immunosuppressive cytokines including IL-10 and TGF-β (39). Foxp3-expressing nTreg cells, which express CD25, lymphocyte activation gene 3 (LAG-3), and CTLA-4, are produced from the thymus and suppress adaptive and innate immune cells (40). ICOS Treg cells are a highly suppressive population of Treg cells which secret IL-10, IFN-γ, and IL-17. It is confirmed that, in human melanoma, ICOS Treg cells are the most suppressive population (41). Tr1 cells, as another Treg subset, can secrete TGF-β at high levels and play an important role in immune tolerance and suppressing autoimmune responses (42, 43). Increasing evidence suggested that the abovementioned regulatory T cells can suppress various effector functions of a wide variety of immune cells such as CD8 T and CD4 cells, NK cells, NKT cells, DCs, and macrophages (40, 44). This is consistent with the GSEA results that few gene sets were distributed in DP T cells and effector memory T cells infiltrated in PCa tissues.

In this study, CD4 naive T cells significantly decreased while Th1 cells and DC cells increased in tumor tissues. We could confirm that CD4 T cells infiltrated in tumors; however, even a higher level of CD4 T cells was found in the central cancer stroma and along the invasive margins of the tumor, which possibly indicated an impeded infiltration into the tumor. DCs are professional antigen-presenting cells (APCs) that can efficiently present antigens to maintain and regulate T cell immune responses in the adaptive immune system (45). In the tumor microenvironment, DCs migrate into tumor tissue through blood circulation and interact with cancer cells and TIICs. However, it seems unlikely that tumor-infiltrating dendritic cells (TIDCs) possess the ability to present tumor-derived antigen *in situ* for antigen presentation, without further TIDCs maturational status (46). The results indicated that cancer cells may affect the antigen presentation ability of DCs, which in turn contribute to the immune escape of tumor cells. From the networks established by the DEGs, we screened the three potential key molecules including CAV1, FLNA, and VCL, which were significantly down-regulated and remarkably associated with PCa patient overall survival. Caveolin-1 (CAV1) is a key organizer of membrane specializations that orchestrate signal transduction and membrane and protein traffic in polarized various cell types. Previous investigations have established CAV1 as vital to T cell function in the context of both antigen presentation and signal transduction (47). A study indicated that CAV1 orchestrates specific T cell responses to Ag receptor engagement, facilitating the polarized redistribution of membrane rafts to the APC-T cell contact in CD8 T cells (48). Knockdown of CAV1 expression resulted in significantly decreased migration and proliferation and impaired ability of cells to form immune synapses. However, over-expression of CAV1 was associated with immune tolerance in chronic lymphocytic leukemia cells (49). Mammalian filamins (FLNs) are a family of three large actin-binding proteins which affect cell spreading and migration. FLN-deficient cells spread to a lesser extent than wild-type or single knockdown cells (50). Vinculin (VCL), an actin filament (F-actin)-binding protein, may play important roles in cell matrix adhesion, cell–cell adhesion, cell morphology, and locomotion (51, 52), indicating that TIIC polarity and synaptic compositions are modulated, which may affect TCR signal transduction and functional output in the TIICs of PCa microenvironment.

In conclusion, this study revealed the distinct immune infiltration patterns of TIICs in PCa and identified several crucial genes that may be involved in membrane polarity of the intratumoral immune cells and play an important role for PCa prognosis. Nevertheless, it reminds us that there are certain limits to the analysis since there are many different gene interactions resulting from various cellular conditions. These exploratory analyses still provide information about potential candidate genes as well as the signal pathways underlying TIICs in TME and bestow a theranostic perspective to the current trend of research.

## Data Availability Statement

Publicly available datasets were analyzed in this study. This data can be found here: TCGA.

## Ethics Statement

The studies involving human participants were reviewed and approved by Affiliated Cancer Hospital and the First Affiliated Hospital of Zhengzhou University. The patients/participants provided their written informed consent to participate in this study. Written informed consent was obtained from the individual(s) for the publication of any potentially identifiable images or data included in this article.

## Author Contributions

SZ, XH, and XQ conceived and designed the study. XH, XQ, and SC conducted data analysis. XH, SZ, and SC wrote and prepared the original draft. XH, SC, and SZ revised the manuscript. All authors reviewed and approved the final manuscript.

## Conflict of Interest

The authors declare that the research was conducted in the absence of any commercial or financial relationships that could be construed as a potential conflict of interest.

## Abbreviations

AUC: area under the curve
APCs: antigen-presenting cells
BC: breast cancer
CAV1: caveolin-1
CTLA4: cytotoxic T lymphocyte antigen 4
DAB: diaminobenzidine
DAVID, Database for Annotation: Visualization and Integrated Discovery
DC: dendritic cells
DEGs: differentially expressed genes
DLX1: distal-less 1
EPC: edge-percolated component
FLNA: mammalian filamins
GO: Gene Ontology
GPX2: glutathione peroxidase 2
GSEA: Gene Set Enrichment Analysis
HOXC6: homeobox protein Hox-C6
ICOS: inducible co-stimulator
IHC: immunohistochemistry
iTreg: inducible regulatory T cells
KEGG: Kyoto Encyclopedia of Genes and Genomes
LAG3: lymphocyte activation gene 3
NK: natural killer
NKT: natural killer T
nTreg: natural regulatory T cells
OS: overall survival
PCa: prostate cancer
PPI: protein–protein interaction
PRAD: prostate adenocarcinoma
ROC: receiver operating characteristic
SCLC: small cell lung carcinoma
TCGA: The Cancer Genome Atlas
Tfh: follicular helper T cells
TIDCs: tumor-infiltrating dendritic cells
TIICs: tumor-infiltrating immune cells
TME: tumor microenvironment
Treg: regulatory T cells
VCL: vinculin.
